# Blood compatibility of widely used central venous catheters; an experimental study

**DOI:** 10.1038/s41598-022-12564-z

**Published:** 2022-05-21

**Authors:** Hulda Thorarinsdottir, Thomas Kander, Dorota Johansson, Bo Nilsson, Bengt Klarin, Javier Sanchez

**Affiliations:** 1 Department of Clinical Sciences, Lund University, Lund, Sweden; 2 Division of Intensive and Perioperative Care, Skane University Hospital, Getingevägen 4, 22185 Lund, Sweden; 3 Bactiguard AB, Tullinge, Stockholm, Sweden; 4 Department of Immunology, Genetics and Pathology, Uppsala University, Uppsala, Sweden; 5 Department of Clinical Sciences, Karolinska Institute, Danderyd Hospital, Sweden and Bactiguard AB, Tullinge, Stockholm, Sweden; 6 Present Address: Ladoroto AB, Stockholm, Sweden

**Keywords:** Analytical biochemistry, Biological models, Immunological techniques

## Abstract

An inserted central venous catheter (CVC) is considered foreign material by the inert host defence systems and induce inflammation and thrombus formation. The objective of this study was to evaluate blood compatibility of six commonly used CVCs. Three coated and three uncoated CVC materials were tested in a modified Chandler loop model. Each catheter material circulated in blood from ten different healthy volunteers for 1 h. Blood cell counts and measurements of the inert host defence systems were performed on blood samples from the loop. All the tested catheters demonstrated impact on blood cells, contact coagulation, the complement system, or inflammatory markers, although the impact varied significantly. Of the catheters we evaluated, the most unfavourable blood compatibility profile was found for the polyurethane CVC coated with chlorohexidine and silver sulfadiazine. The greatest variation in blood compatibility between test runs was noted for the silicone dialysis catheter. Poor blood compatibility should be taken seriously but given the experimental design of the current study the clinical significance remains to be evaluated.

## Introduction

The use of central venous catheters (CVCs) is essential in patient care. Two major complications associated with the use of these devices are catheter-related bloodstream infections (CRBSIs) and catheter-related thrombosis, which are consequences of the catheter breaking the body’s natural defence barriers and the host defence targeting the foreign CVC material.

**Figure 1:**
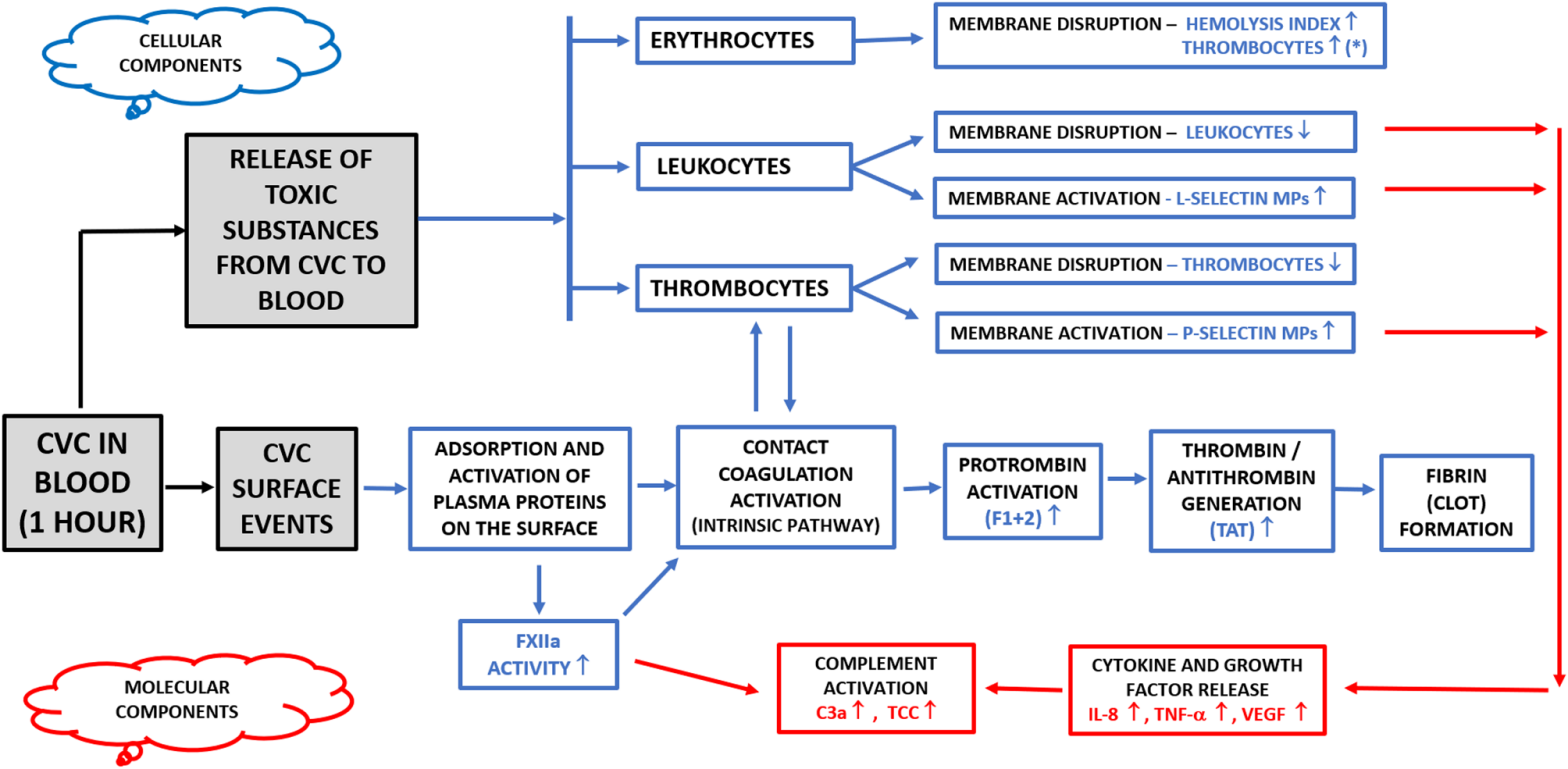
Activation of host defences on response of insertion of a central venous catheter (CVC).When a foreign material such as a CVC is inserted into the bloodstream, various defense systems aimed at eliminating the foreign material are activated in the blood, including blood cells, contact coagulation, the complement system, and inflammation. *False increase in thrombocytes when fragments of erythrocytes and leukocytes membrane interfere with analysis of much smaller thrombocytes.

When a CVC is inserted into the bloodstream, the catheter surface is almost immediately covered with a layer of plasma proteins, which changes the surface characteristics of the material^1,2^. Subsequent activation of the host’s defences can induce inflammation and thrombus formation, depending on the composition and the activation mechanisms of the proteins adsorbed on the surface of the catheter (Fig. 1)^3^. Initially, inflammatory mediators are generated on the biomaterial surface, subsequently they can spread in plasma via soluble activation products, activated leukocytes, and platelets and thereby lead to whole-body inflammation^4,5^.

As the catheter surface material plays an important role in the pathogenesis of infection and thrombus formation, modifying the CVC itself by use of different types of impregnation or coating has been applied in efforts to reduce CRBSIs and catheter-related thrombosis^6^. To date, CVCs coated with chlorohexidine silver sulfadiazine (CHSS) or minocycline-rifampicin are the most widely investigated and have shown significantly lowered microbial colonization rates and a decrease in CRBSIs^6–8^, although their overall benefits in reducing clinical sepsis and mortality remain uncertain^9^. To the best of our knowledge, few studies have evaluated these devices regarding the risk of thrombosis and inflammation, an aspect that would be of interest, considering that the mentioned CVCs release antimicrobial substances that are potentially toxic. Various trials have assessed the use of other substances such as silver, heparin, and benzalkonium to impregnate CVCs. This is illustrated by a large meta-analysis showing that silver-impregnated CVCs are effective in reducing CRBSIs^9^. Benzalkonium-impregnated CVCs have not been studied as thoroughly but have nonetheless been found effective in reducing bacterial colonization of the catheter^9^.

In an attempt to further describe differences in blood compatibility between various CVC materials we designed the present study with the aim to evaluate the activation of hemolysis, coagulation, the complement system, and inflammation of three coated and three uncoated catheter materials, extensively used in clinical praxis.

## Materials and methods

The study protocol was approved by the Regional Ethical Review Board, Stockholm, Sweden (protocol 2010/1627-31/3). Prior to entering the investigation, ten healthy volunteers who had not taken any medications during the 15 days prior to blood donation signed informed consent. All procedures and analyses were carried out in accordance with relevant guidelines and regulations.

### CVC material description

The venous access materials are described in Table 1. Five CVCs and one hemodialysis catheter (CHC) were selected for this ex vivo laboratory study. The three uncoated materials included two CVCs made of polyurethane (PU) and one CHC made of silicone, all of which are used extensively in Europe. For comparison, we selected three CVCs made of PU with the following anti-infective coatings: (1) a combination of chlorhexidine and silver sulfadiazine; (2) a combination of benzalkonium chloride in a hydrophilic matrix (hydromer); and (3) a noble metal alloy comprising silver, gold, and palladium. Coated CVCs are widely used in the United States. All five CVCs in our study had three injection ports (triple lumen) and were 2.3 mm in diameter (7 Fr). The CHC had two injection ports (double lumen) and was 4.3 mm in diameter (13 Fr).

**Table 1:** Description of catheter materials.

Type	Brand	Abbreviation	Material(s)
Uncoated	MedComp, CHC, Hemo-cath ST	Si-1	Silicone
	MeritMedical, CVC, Careflow®	PU-1	Polyurethane
	Teleflex, CVC, Arrow MultiLumen CVC with Blue Tip®	PU-2	Polyurethane
Coated	Teleflex, CVC, ARROWg + ard Blue® with Blue Tip	PU-2 + CHSS	Polyurethane coated with chlorohexidine and silver sulfadiazine
	Argon Medical, CVC, Hydrocath Assure™	PU-3 + BZC	Polyurethane with a hydrophilic matrix impregnated with benzalkonium chloride
	Bactiguard, CVC, Infection Protection Central Venous Catheter	PU4 + NbMC	Polyurethane coated with noble metals (Pd, Au, and Ag)

*CVC* Central venous catheter, *CHC* Hemodialysis catheter, *Pd* Palladium; *Au* Gold, *Ag* Silver.

### Blood collection, preparation, and the experimental phase

**Figure 2:**
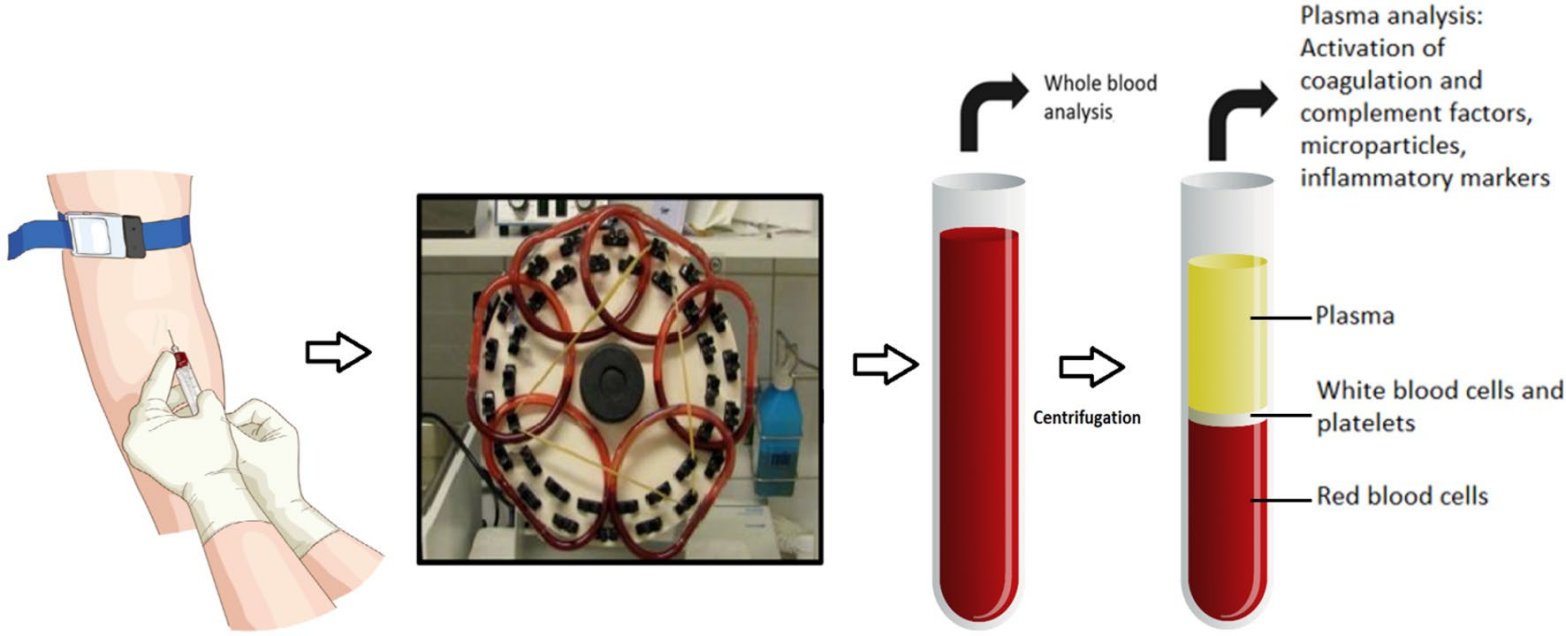
The Chandler loop model. The model imitates the flow of blood in a vein.

The previously described modified Chandler loop model available at Danderyd Hospital (Danderyd, Sweden), was used to evaluate blood compatibility^10^, Fig. 2. On each test occasion, a 35-mL blood sample was collected from each of the ten healthy blood donor and heparin added to the collection tube with a final concentration of 0.1 IU/mL. A 4.5-mL aliquot of the sample was poured into each of the loops, and a 1.4-cm^2^ piece of each of the tested catheter materials was put in the separate loops. One loop contained blood but no catheter material to serve as a control (designated the “control loop”). The material samples were circulated in the loops for 1 h to imitate the flow of blood in a vein. Thereafter, the pieces of the materials were removed from the blood and an aliquot of 2.7 mL blood was collected from the loops and either EDTA (BD Vacutainer^®^, K2E 5.4 mg, used for all assessments except microparticle analysis) or citrate (BD Vacutainer^®^, 9NC 0.129 M, used for microparticle analysis) was added to stop any ongoing activation of blood components. Finally, the blood samples were centrifuged at 2000 × g for 20 min at room temperature. The supernatant was then recentrifuged, at 13,000 × g for 2 min at room temperature. The plasma was stored at − 70 °C.

### Blood compatibility assays

Blood compatibility was evaluated using parameters related to the activation of hemolysis, coagulation, the complement system, and inflammation. The parameters were chosen according to ISO standard 10993-4: Biological evaluation of medical devices, Part 4: Selection of tests for interaction with blood. All blood compatibility tests were analyzed at the Clinical Research center (*Kliniskt forskningscentrum*) at Danderyd Hospital, Danderyd, Sweden.

### Activation of hemolysis and coagulation

Hemolysis was analyzed using a QuantiChrome™ Hemoglobin assay kit (Bioassay Systems, Hayward, CA, USA) to evaluate free hemoglobin present in plasma due to rupture of red blood cells. The hemolysis index was corrected according to international standard F756-17. To determine whether the extensive hemolysis caused by the PU-2 + CHSS material might be due to activation of the complement system, a 1-cm-long piece of a PU-2 + CHSS catheter was exposed to 1 mL of blood containing the complement inhibitor eculizumab (50 mcg/mL) for 30, 60, or 120 min. EDTA blood was used as a control with the same incubation time.

Prothrombin fragment 1 + 2 (F1 + 2) and thrombin-anti-thrombin complex (TAT) were measured in the EDTA sample by using a commercially available ELISA kit (Enzygnogst^®^ F1 + 2 [Monoclonal]) and Enzygnost^®^ TAT micro (Siemens AG, Munich, Germany) according to the instructions of the manufacturer. Factor XII surface activity was evaluated as follows: 0.5 cm of each material was incubated with normal pool plasma for 15 min and then washed three times with Tris buffer; next, each material was incubated with 100 μL of an FXII-deficient plasma (Hageman plasma) diluted 1:500 in Tris buffer for 10 min, after which 100 uL of chromogenic reagent (S-2302) was added, and the sample was further incubated for 5 min; the reaction was stopped with 100 μL of citric acid (20%), and 200 μL of each sample was placed on a microtiter plate and absorbance was read at 405 nm.

### Complement activation and acute immune reaction

Complement activation (C3a and sC5b-9 [terminal complement complex]) in the EDTA samples was evaluated using a C3a and sC5b-9 Plus EIA Kit (San Diego, CA, USA) according to the instructions of the manufacturer. Levels of interleukin 8 (IL-8), tumor necrosis factor alpha (TNF-α), and vascular endothelial growth factor (VEGF) were measured in EDTA plasma samples by using the electro-chemiluminescence immunoassay plates V-PLEX Plus Proinflammatory Panel 1 and V-PLEX Plus Human VEGF kit (Meso Scale Discovery’s Multi Array^®^ [MSD, Rockville MD, USA]), and a sector imaging S2400 instrument (MSD), as stipulated by the manufacturer.

### Analysis of P-Selectin (CD62-P) and L-Selectin (CD62-L) microparticles

Microparticles (MPs) released from platelets (P-selectin) and leukocytes (L-selectin) were quantified after ultracentrifugation of the citrate-diluted samples as previously described^11^. The analysis was performed in a Beckman Coulter Gallios™ flow cytometer using Bovine Lactaderin-FITCH (Haematologic Technologies Inc), CD62L-APC, and CD62-P (Beckman Coulter, France). To determine the MP gate, Sphero™ AccuCount Particles (ACBP-20-10 Nordic BioSite AB) was used to exclude cell membrane fragments from P- and L-selectin. The MP concentration was calculated as follows: (MPs counted × standard beads added/L)/standard beads counted.

### Statistics

Sample size calculation for the primary endpoint (hemolysis) was based on previously reported data^10^ for the two tailed Wilcoxon’s signed rank sum test. Given a power of 0.80 and an alpha level of 0.008, a sample size of 9 was required. The results of blood compatibility tests were expressed as median (minimum to maximum range). When comparing blood compatibility variables, we used non-parametric tests for dependent samples (Friedman test and Wilcoxon’s signed rank sum test), because each blood donor served as his or her own control. Corrections for multiple testing were performed by Bonferroni correction for the primary outcome so that *P* < 0.0083 was considered significant. For the secondary outcomes *P* < 0.05 was considered significant only if all ranks in the hypothesis testing between materials were pointing in same direction. All statistical tests were two-tailed, and all analyses were performed using SPSS 24 (SPSS Inc., Chicago, IL, USA). *P* < 0.05 was considered significant.

## Results

Detailed results of the blood compatibility tests for the six catheter materials and the control run in the Chandler loop model are demonstrated in Tables 2 and 3. *P* values from multiple comparisons are presented in Supplementary Table S1a and b.

**Table 2:**
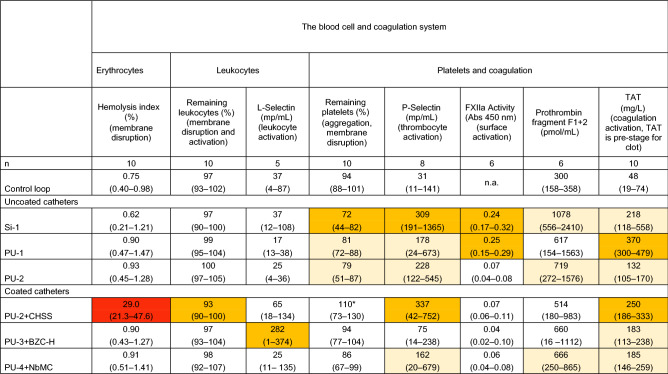
Blood compatibility tests for the six catheter materials and the control loop after circulation in the Chandler loop system.

Results are shown as median (min–max range). Colors: light orange, significantly different from control loop; orange, significantly different from control loop and at least one other CVC material; red, significantly different from control loop and all other CVC materials. *This catheter showed a higher platelet count after exposure to the tested material than in fresh blood (110%) because fragments of disrupted erythrocytes and leukocytes interfered with the platelet measurements, resulting in a false high platelet count.

**Table 3:**
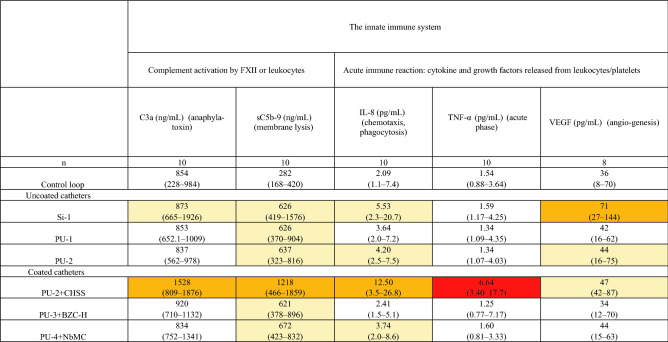
Blood compatibility tests for the six catheter materials and the control loop after circulation in the Chandler loop system.

Results are shown as median (min–max range). Colors: light orange, significantly different from control loop; orange, significantly different from control loop and at least one other CVC material; red, significantly different from control loop and all other CVC materials.

### Blood cell system

#### Erythrocytes

##### Hemolysis index

According to the international standard ASTM F756-17, a hemolytic index of > 5% is classified as “hemolytic.” All catheters except PU-2 + CHSS showed “non-hemolytic” properties, with median hemolytic indexes ranging from 0.6 to 0.9%. Blood exposed to the PU-2 + CHSS material had a median hemolytic index of 29%, which is significantly higher than the rates noted for the other five materials and the control loop (*P* = 0.005 for all comparisons). To determine whether the hemolysis was due to complement activation, we exposed the PU-2 + CHSS material to the C5 inhibitor eculizumab (50ug/mL), which prevents formation of the membrane attack complex and complement-mediated lysis. However, the hemolytic index was still 27–36%, indicating that hemolysis was not due to complement activation but rather to a toxic effect of the PU-2 + CHSS coating.

#### Leukocytes

##### Remaining leukocytes

Membrane disruption of leukocytes leads to a decrease in the count of such cells in a blood analysis. The leukocytes remaining after circulating in the Chandler loop system ranged from 93 to 100% in plasma exposed to the six catheter materials and the control loop. Only blood exposed to PU-2 + CHSS showed a significantly lower level of remaining leukocytes compared to the control loop. Also, PU-2 + CHSS exposure led to a lower rate of remaining leukocytes compared to all the other materials except Si-1.

##### L-selectin MPs

Activation of leukocyte membranes causes a release of the cell adhesion protein L-selectin MPs (CD62L) from the surface of the platelets. The median number of L-selectin MPs ranged from 17/mL to 282/mL in plasma exposed to the six catheter materials and 37/mL in the control loop. The release of MPs was significantly higher (282/mL) in plasma exposed to the PU-3 + BZC material than in the control loop. When comparing the different materials, PU-3 + BZC induced significantly higher L-selectin levels than PU-2 and PU-4 + NbMC.

#### Platelets and coagulation

##### Remaining platelets

Both the disruption of platelet membranes and the aggregation of platelets causes a decrease in platelet counts after circulation in the Chandler system. The median number remaining platelets ranged from 72 to 94% for all materials except PU2 + CHSS; blood exposed to this catheter showed falsely higher platelets counts of 110% due to the presence of fragments of disrupted erythrocytes and leukocytes interfering with the platelet measurements. The uncoated catheters (Si-1, PU-1, and PU-2) all showed significantly lower levels of remaining platelets than the control loop. When comparing the tested materials, Si-1 resulted in significantly lower levels of remaining platelets than all the other materials except PU-2.

##### P-selectin MPs

Activation of platelet membranes causes release of the cell adhesion glycoprotein, P-selectin MPs (CD62P) from the surface of the platelets. The median number of P-selectin MPs ranged from 75/mL to 337/mL in plasma exposed to the six catheter materials and 31/mL in the control loop. The P-selectin levels were significantly higher in plasma exposed to all the materials except PU3 + BZC in comparison with the control loop. When comparing the materials, PU-2 + CHSS and Si-1 showed significantly higher levels of P-selectin MPs than all the other materials except PU-2. 

##### FXIIa activity

Factor XII (FXII) is the first of the plasma proteins to be activated by contact with a foreign material present in the blood, resulting in formation of FXIIa. The median activity of FXIIa (absorbance at 405 nm) ranged from 0.04 to 0.25 on the surface of the six catheter materials tested in our study. Measurement of FXIIa was not applicable in the control loop, because FXII is activated by the surface of a catheter. Upon exposure to plasma, FXIIa showed significantly higher activity on the surfaces of Si-1 (0.24) and PU-1 (0.25) compared to all other materials.

##### Prothrombin fragment F1 + 2

Prothrombin fragment F1 + 2 is a peptide released from prothrombin during its activation. The median generation of F1 + 2 ranged from 514 to 1078 pmol/mL in plasma exposed to the six catheter materials and was 300 pmol/mL in the control loop. The Si-1, PU-2, and PU4 + NbMC materials all resulted in significantly higher levels of F1 + 2 than noted in the control loop. We found no significant difference in generation of F1 + 2 between the six materials (*P* = 0.071).

##### TAT

TAT is an indicator of activation of the coagulation cascade and is generated when thrombin neutralizes anti-thrombin. The median generation of TAT in the blood in contact with the six catheter materials ranged from 132 to 370 µg/L and was significantly higher than the low level of 48 µg/L observed in the control loop. The PU-1 catheter led to the highest TAT level of 370 µg/L, which was significantly higher than compared to all the other materials except Si-1. PU-2 + CHSS resulted in a TAT level of 251 µg/L, which was significantly higher than the levels noted for PU-2, PU-3 + BZC, and PU-4 + NbMC.

### Molecular markers of the innate immune system

#### Activation of the complement system

Activation of the complement system via the cellular system (leukocytes and platelets) or via protein absorption on the material surface was assessed by analyzing the increase in inflammatory anaphylatoxin C3a and soluble terminal complement complex sC5b-9, a marker of cell membrane lysis, after exposure to the six catheter materials and the control loop.

##### C3a anaphylatoxin

The median generation of C3a ranged from 834 to 1528 ng/mL in plasma exposed to the six catheter materials and was 854 ng/mL in the control loop. The PU-2 + CHSS and Si-1 materials generated significantly higher C3a compared to the control loop. The median value for Si-1 was low (873 ng/mL) but varied markedly (range 665–1926 ng/mL), resulting in statistical significance in paired comparisons. The PU-2 + CHSS catheter generated the highest C3a levels (1528 ng/mL), which were significantly higher than compared to the control loop and all the materials except Si-1.

##### sC5b-9 marker

The results of the analysis of sC5b-9 showed a trend similar to that observed in the C3a analysis, which was expected. As for C3a, the PU-2 + CHSS catheter generated the highest median level of sC5b-9 of 1218 ng/mL, which was significantly higher than noted for all the other materials except Si-1. In contrast to C3a, all catheters generated significantly higher levels of sC5b-9 than the control loop.

#### Acute inflammatory reaction: IL-8, TNF-α, and VEGF

To assess the acute inflammatory reaction caused by leukocyte and thrombocyte activation or membrane disruption, we analyzed IL-8, TNF-α, and VEGF released from leukocytes into plasma upon exposure to the six different catheter materials and the control loop.

##### Interleukin 8

The median generation of IL-8 was 2.09 pg/mL in the control loop and ranged from 2.41 to 12.5 pg/mL after exposure to the six catheter materials. Compared to the control loop, the Si-1, PU-2, PU2 + CHSS, and PU4 + NbMC materials generated significantly higher IL-8 levels. Also, PU-2 + CHSS generated significantly higher IL-8 than all the other materials except Si-1.

##### TNF-α

The median level of TNF-α was 1.54 pg/mL in the control loop and ranged from 1.25 to 6.64 pg/mL after exposure to the six catheter materials. The PU-2 + CHSS catheter generated significantly higher TNF-α than noted for the control loop and all the other materials.

##### VEGF

Median generation of VEGF was 36 pg/mL in the control loop and ranged from 33 to 71 pg/mL after exposure to the six catheter materials. Compared to the control loop, the Si-1 material, PU-2, and PU-2 + CHSS catheters generated significantly higher VEGF levels. The Si-1 generated the highest VEGF levels, which were significantly higher than the levels induced by PU-1, PU-2, PU-3 + BZC, and PU-4 + NbMC but not PU-2 + CHSS.

## Discussion

In this experimental study all six tested catheter material from commonly used central venous accesses activated coagulation, the complement system, and inflammation to some extent, but there were significant differences between the devices. The polyurethane catheter coated with chlorohexidine and silver sulfadiazine exhibited reduced blood compatibility including increased hemolysis and inflammation, compared to the other catheters. The silicone catheter showed the greatest variation in blood compatibility test results. Poor blood compatibility could cause inflammation and facilitate the development of catheter-related thrombosis in patients receiving these central venous catheters, but given the experimental design of the current study, clinical significance has to be studied further.

### Blood coagulation and inflammation in the presence of foreign materials

Entry of a foreign CVC material into the bloodstream triggers hemostatic processes (Fig. 1). Surface-related events depend on the structure and/or chemistry of the surface in question, and they initially involve adsorption and the activation of plasma proteins (see Fig. 1). FXII is the first protein to be activated in the contact activation system. The resulting FXIIa initiates contact coagulation activation, generation of thrombin (measured as TAT level), and fibrin clot formation. Complement is also activated by other mechanisms, such as the adsorption of immunoglobulins, Ficolin-2, and C3 (leading to increased C3a and sC5b-9). FXIIa can also trigger complement activation via C1s. Given the poor ex vivo blood compatibility demonstrated in the present study it is surprising how well the studied catheter materials seem to be tolerated in vivo^8,12,13^. However, it should be noted that any symptoms originating from bad blood compatibility from the catheter material may be masked by the patient’s critical condition given the need of a central venous access.

### Differences between the tested materials

Compared to the control loop, all the materials assessed in this study to some extent triggered either the blood cell system or the molecular innate immune system when tested in the Chandler model (Fig. 1 and Tables 2 and 3).

Among the uncoated catheter materials, silicone (Si-1) demonstrated the lowest blood compatibility and also the greatest variation in such compatibility between test runs. Silicone seemed to trigger contact coagulation, as indicated by increased levels of FXIIa, prothrombin fragment F1 + 2, and TAT, leading to platelet activation and aggregation with the release of P-selectin MPs and VEGF. Activation of both the complement system and inflammation was also demonstrated by increased levels of C3a, sC5b-9, and IL-8. The activation of contact coagulation and thrombocytes by silicone materials has been observed in previous studies in which modifications of the silicone surface were found to result in improved blood compatibility^14,15^. A potential mechanism for this effect is that the negative charge of the silicone surface activated not only FXII, but also thrombocytes, via the intrinsic contact coagulation pathway^16,17^. The pronounced variation in surface properties between test runs might be explained by different amounts of silicone oil slowly exuding to the surface of the material during storage, thereby renewing its negative charges. This problem has been observed in studies of silicone breast prostheses^18^. Such exudation of silicone oil is also potentially toxic to thrombocytes, representing the same type of negative effect^14^. In animal studies, compared to polyurethane catheters, silicone catheters have been found to show increased susceptibility to staphylococcal infections and a stronger local inflammatory response^19,20^. These effects have been demonstrated to be secondary to increased complement activation via the alternative pathway, thus leading to reduced opsonizing ability and increased risk of infection^21^. Our results corroborate those findings but also further describe the different mechanisms that are activated when silicone comes in contact with blood, Tables 2 and 3.

The two polyurethanes PU-1 and PU-2 differed significantly in blood compatibility, indicating variation in the surface composition of polyurethanes from different manufacturers. PU-2 exhibited good blood compatibility with only a slight increase in P-selectin MPs, F1 + 2, TAT, and complement activation compared to the control loop (Tables 2 and 3). PU-1, on the other hand, induced contact coagulation (increased FXIIa and TAT), which can theoretically increase the risk of catheter-related thrombosis^13,22^. Differences in the composition of the two polyurethane catheters may include the presence of sulphate groups, which would give the surface a negative charge and thereby trigger FXII and activation of contact coagulation (i.e. increased TAT is seen)^23^.

It is also possible that the surface of a catheter coated with an anti-infective substance may impair the blood compatibility of the device. In the present study, this particularly applies to the polyurethane catheter coated with chlorhexidine and silver sulfadiazine (PU-2 + CHSS), which demonstrated the most unfavorable blood compatibility in this ex vivo laboratory study by inducing substantial hemolysis, platelet activation, TAT, F1 + 2 generation, complement activation, and release of proinflammatory cytokines. The effectiveness of the PU-2 + CHSS catheter in reducing CRBSI has been extensively investigated^9,12,24^, and international guidelines recommend the use of this catheter to reduce CRBSIs in critically ill patients^25^. To the best of our knowledge, thrombotic or hemolytic complications associated with the use of the PU-2 + CHSS catheters have not been observed in clinical studies. Animal experiments have demonstrated that chlorhexidine diacetate in a range of concentrations is damaging to rabbit erythrocytes^26^ as it binds to phospholipids in the cell membrane causing rupture of the cell and release of fractions of cell membrane and heme into the blood. Accordingly, as seen in our study, fragments of disrupted erythrocytes and leukocytes interfered with the platelet measurements, giving rise to falsely higher platelets counts of 110%. Further, our results indicate that the hemolysis in the PU-2 + CHSS loop (29%) was due to a toxic effect of the coating rather than primary complement activation, as the hemolysis occurred after complement inhibition with eculizumab or calcium. The strong complement activation seen with PU-2 + CHSS catheters might be explained by massive leakage of heme into plasma, which is known to activate the complement system^27^.

Chlorhexidine is a commonly used synthetic antiseptic and disinfectant that was introduced in the 1950s, and it affects both gram-negative and gram-positive bacteria, and also Candida albicans and some viruses^28^. The use of chlorhexidine in healthcare settings has increased substantially in recent years, and the results of large studies have indicated that this might lower the rates of healthcare-associated infections in different settings^29,30^. Case reports have described allergic reactions associated with use of chlorhexidine-impregnated CVCs in several countries^31–34^. It appears that the prevalence of allergic reactions is greater when chlorhexidine comes in contact with mucosal membranes or blood as compared to the skin, especially in persons of Japanese descent^29,30,35,36^, although more recent case reports describe allergic reactions in other ethnic groups as well^33^. Few investigations have focused on the systemic effects of chlorhexidine, but there are some case reports describing chlorhexidine as highly toxic and causing acute respiratory distress syndrome and shock^35,37^.

The release of proinflammatory cytokines has been associated with an increased risk of venous thrombosis, but the contribution of each cytokine remains to be elucidated^38,39^. Both silver sulfadiazine and chlorhexidine are known allergens^36,40^, which is consistent with the activation of pro-inflammatory markers seen in our study. Interestingly, Staphylococcal species, the most common catheter-infecting organisms, show receptor-mediated binding with fibronectin, fibrin, and other components of the fibrin sheath^3,41^. Hence, it is possible that impaired blood compatibility and limited duration of the coating (< 15 days in laboratory studies)^42^ can partly explain why it appears that antimicrobial CVCs do not clearly reduce clinically diagnosed sepsis or mortality^9^.

The second coated CVC we tested, PU-3 + BZC-H, was coated with both the cationic surfactant benzalkonium chloride and a hydrophilic hydromer, the latter of which is intended to improve the blood compatibility of the surface. All blood compatibility parameters except L-selectin were found at low levels. It is possible that the release of L-selectin from leukocytes can be triggered by a toxic effect of benzalkonium chloride, as has been suggested by other researchers, and this may potentially have a negative impact on the patients’ immune defense^43^.

It should also be noted that, compared to the control loop, the CVC coated with a noble metal alloy, PU-4 + NbMC, led to a slight increase in P-selectin MPs, F1 + 2, TAT, and complement activation. This noble metal coating has previously been evaluated in an in vitro blood compatibility study^10^, although the reporting authors described fewer blood compatibility parameters and values differing from those we noted (a TAT level of ~ 300 µg/L compared to ~ 200 µg/L in our study). Those investigators also described similar behavior in contact with blood as observed in our study.

### Limitations of an experimental model and possible application to an in vivo situation

We recognize the limitations of the present study given the experimental design. However, the method we used is well established and recognized for testing of materials used in health care contexts and can elucidate relative differences in blood compatibility between CVC materials. It should be noted that the experimental set up in the present study may facilitate the accumulation of any released chlorohexidine from the coated surface which may have affected the results of the blood combability tests which may not be as obvious in the clinical setting where released chlorohexidine is diluted in a large blood volume. Nevertheless, blood combability may be an issue with the PU-2 + CHSS. In this experimental study the exposure of material surface to blood volume was 140 times higher per hour compared to an in vivo situation. In about 6 days the exposure in vivo would be the same as in the experiment. Prior data show that patients have their CVC about a week on average^44^. Accordingly, the clinical significance remains to be explored and may also vary for different patient groups depending on their diseases, comorbidities and medication with anti-inflammatory or anticoagulant therapy. Our investigation was conducted using a limited number of catheters from one or two different product lots, and possible variability between lots has not been evaluated.

## Conclusion

In this experimental study all catheter material from six commonly used central venous accesses activated coagulation, the complement system, and inflammation to some extent, but there were significant differences between the devices. The polyurethane catheter coated with chlorohexidine and silver sulfadiazine exhibited reduced blood compatibility including increased hemolysis, compared to the other catheters. The silicone catheter showed the greatest variation in blood compatibility test results. Poor blood compatibility should be taken seriously but given the experimental design of the current study the clinical significance remains to be evaluated.

## Supplementary Information

Supplementary Information.

## Data Availability

The datasets used and/or analysed during the current study are available from the corresponding author on reasonable request.

## References

[1] Borow M, Crowley J (1986). Prevention of thrombosis of central venous catheters. J. Cardiovasc. Surg. (Torino).

[2] Mermel LA (2009). Clinical practice guidelines for the diagnosis and management of intravascular catheter-related infection: 2009 Update by the Infectious Diseases Society of America. Clin. Infect. Dis..

[3] Horbett TA (2018). Fibrinogen adsorption to biomaterials. J. Biomed. Mater. Res. A.

[4] Ekdahl KN, Soveri I, Hilborn J, Fellström B, Nilsson B (2017). Cardiovascular disease in haemodialysis: Role of the intravascular innate immune system. Nat. Rev. Nephrol..

[5] Nilsson B, Ekdahl KN, Mollnes TE, Lambris JD (2007). The role of complement in biomaterial-induced inflammation. Mol. Immunol..

[6] Lai, N. M. *et al.* Catheter impregnation, coating or bonding for reducing central venous catheter‐related infections in adults. *Cochrane Database Syst. Rev.* (2016). 10.1002/14651858.CD007878.pub3 PMC6517176 26982376

[7] Hanna H (2004). Long-term silicone central venous catheters impregnated with minocycline and rifampin decrease rates of catheter-related bloodstream infection in cancer patients: A prospective randomized clinical trial. J. Clin. Oncol..

[8] Wang H (2018). Effectiveness of antimicrobial-coated central venous catheters for preventing catheter-related blood-stream infections with the implementation of bundles: A systematic review and network meta-analysis. Ann. Intens. Care.

[9] Chong HY, Lai NM, Apisarnthanarak A, Chaiyakunapruk N (2017). Comparative efficacy of antimicrobial central venous catheters in reducing catheter-related bloodstream infections in adults: Abridged Cochrane systematic review and network meta-analysis. Clin. Infect. Dis..

[10] Vafa Homann M, Johansson D, Wallen H, Sanchez J (2016). Improved ex vivo blood compatibility of central venous catheter with noble metal alloy coating. J. Biomed. Mater. Res. Part B Appl. Biomater..

[11] Mobarrez F (2012). Release of endothelial microparticles in vivo during atorvastatin treatment; a randomized double-blind placebo-controlled study. Thromb. Res..

[12] Brun-Buisson C (2004). Prevention of intravascular catheter-related infection with newer chlorhexidine-silver sulfadiazine-coated catheters: A randomized controlled trial. Intens. Care Med..

[13] Nurdin N (2003). Haemocompatibility evaluation of DLC-and SiC-coated surfaces. Eur. Cell Mater..

[14] Li M (2012). Surface modification of silicone for biomedical applications requiring long-term antibacterial, antifouling, and hemocompatible properties. Langmuir.

[15] Melvin ME, Fissell WH, Roy S, Brown DL (2010). Silicon induces minimal thromboinflammatory response during 28-day intravascular implant testing. ASAIO J..

[16] Arvidsson S, Askendal A, Tengvall P (2007). Blood plasma contact activation on silicon, titanium and aluminium. Biomaterials.

[17] Steuer H, Krastev R, Lembert N (2014). Metallic oxide nanoparticles stimulate blood coagulation independent of their surface charge. J. Biomed. Mater. Res. Part B Appl. Biomater..

[18] Lykissa ED, Kala SV, Hurley JB, Lebovitz RM (1997). Release of low molecular weight silicones and platinum from silicone breast implants. Anal. Chem..

[19] Mehall JR, Saltzman DA, Jackson RJ, Smith SD (2002). Fibrin sheath enhances central venous catheter infection. Crit. Care Med..

[20] Sherertz RJ (1995). Contribution of vascular catheter material to the pathogenesis of infection: The enhanced risk of silicone in vivo. J. Biomed. Mater. Res..

[21] Marosok R, Washburn R, Indorf A, Solomon D, Sherertz R (1996). Contribution of vascular catheter material to the pathogenesis of infection: Depletion of complement by silicone elastomer in vitro. J. Biomed. Mater. Res..

[22] Brash JL, Horbett TA, Latour RA, Tengvall P (2019). The blood compatibility challenge. Part 2: Protein adsorption phenomena governing blood reactivity. Acta Biomater..

[23] Gorbet M (2019). The blood compatibility challenge. Part 3: Material associated activation of blood cascades and cells. Acta Biomater..

[24] Casey AL, Mermel LA, Nightingale P, Elliott TS (2008). Antimicrobial central venous catheters in adults: A systematic review and meta-analysis. Lancet Infect. Dis..

[25] O’Grady NP (2011). Guidelines for the prevention of intravascular catheter-related infections. Clin. Infect. Dis..

[26] Ansel HC (1967). Hemolysis of erythrocytes by antibacterial preservatives IV. Hemolytic activity of chlorhexidine diacetate. J. Pharm. Sci..

[27] Merle NS (2018). Intravascular hemolysis activates complement via cell-free heme and heme-loaded microvesicles. JCI Insight.

[28] Karpiński T, Szkaradkiewicz A (2015). Chlorhexidine–pharmaco-biological activity and application. Eur. Rev. Med. Pharmacol. Sci..

[29] Climo MW (2013). Effect of daily chlorhexidine bathing on hospital-acquired infection. N. Engl. J. Med..

[30] Huang SS (2019). Chlorhexidine versus routine bathing to prevent multidrug-resistant organisms and all-cause bloodstream infections in general medical and surgical units (ABATE Infection trial): A cluster-randomised trial. Lancet.

[31] Stephens R (2001). Two episodes of life-threatening anaphylaxis in the same patient to a chlorhexidine–sulphadiazine-coated central venous catheter. Br. J. Anaesth..

[32] Terazawa E (1998). Anaphylactic shock associated with a central venous catheter impregnated with chlorhexidine and silver sulfadiazine. Masui.

[33] Toomey, M. Preoperative chlorhexidine anaphylaxis in a patient scheduled for coronary artery bypass graft: A case report. *AANA J.* **81** (2013). 23923672

[34] Weng M, Zhu M, Chen W, Miao C (2014). Life-threatening anaphylactic shock due to chlorhexidine on the central venous catheter: a case series. Int. J. Clin. Exp. Med..

[35] Ishigami S (2001). Intravenous chlorhexidine gluconate causing acute respiratory distress syndrome. J. Toxicol. Clin. Toxicol..

[36] Ohtoshi T (1986). IgE antibody-mediated shock reaction caused by topical application of chlorhexidine. Clin. Exp. Allergy.

[37] Hirata K, Kurokawa A (2002). Chlorhexidine gluconate ingestion resulting in fatal respiratory distress syndrome. Vet. Hum. Toxicol..

[38] Gao Q (2016). The correlation analysis of tumor necrosis factor-alpha-308G/A polymorphism and venous thromboembolism risk: A meta-analysis. Phlebology.

[39] Matos MF (2011). The role of IL-6, IL-8 and MCP-1 and their promoter polymorphisms IL-6-174GC, IL-8-251AT and MCP-1-2518AG in the risk of venous thromboembolism: A case-control study. Thromb. Res..

[40] de la Hoz Caballer B (1991). Management of sulfadiazine allergy in patients with acquired immunodeficiency syndrome. J. Allergy Clin. Immunol..

[41] Herrmann M (1988). Fibronectin, fibrinogen, and laminin act as mediators of adherence of clinical staphylococcal isolates to foreign material. J. Infect. Dis..

[42] de Sousa JKT, Haddad JPA, de Oliveira AC, Vieira CD, dos Santos SG (2019). In vitro activity of antimicrobial-impregnated catheters against biofilms formed by KPC-producing Klebsiella pneumoniae. J. Appl. Microbiol..

[43] Berg Ø, Bakken A, Steinsvåg S, Farstad M (1999). Benzalkonium chloride interferes with energy production, secretion and morphology in human blood platelets. Platelets.

[44] Thorarinsdottir HR, Rockholt M, Klarin B, Broman M, Fraenkel CJ, Kander T (2020). Catheter-related infections: A Scandinavian observational study on the impact of a simple hygiene insertion bundle. Acta Anaesthesiol. Scand..

