# Supplementary material for: Blood compatibility of widely used central venous catheters; an experimental study
Source: Sci Rep. 2022 May 21;12:8600. doi: 10.1038/s41598-022-12564-z (PMC9124179; doi:10.1038/s41598-022-12564-z)
Supplement: Supplementary file 1 — Supplementary Information. [file 41598_2022_12564_MOESM1_ESM.pdf]

Supplement 1a. Comparisons of the different catheter materials to the control loop and between the six different catheter materials.

The blood cell system

Erythrocytes

Hemolysis index:

	Control loop	Si-1	PU-1	PU-2	PU-2+CHSS	PU-3+BZC-H	PU-4+NbMC
**Control loop**	x						
**Si-1**	<5% hemolysis						
**PU-1**	<5% hemolysis						
**PU-2**	<5% hemolysis						
**PU-2+CHSS**	>5% hemolysis	0.005	0.005	0.005	x	0.005	0.005
**PU-3+BZC-H**	<5% hemolysis						
**PU-4+NbMC**	<5% hemolysis						

Leukocytes

Remaining leukocytes:

	Control loop	Si-1	PU-1	PU-2	PU-2+CHSS	PU-3+BZC-H	PU-4+NbMC
**Control loop**	x						
**Si-1**	0.063						
**PU-1**	0.091						
**PU-2**	0.176						
**PU-2+CHSS**	0.050	0.499	0.021*	0.012	x	0.008	0.013*
**PU-3+BZC-H**	0.138						
**PU-4+NbMC**	0.779						

L-selectin MPs

	Control loop	Si-1	PU-1	PU-2	PU-2+CHSS	PU-3+BZC-H	PU-4+NbMC
**Control loop**	x						
**Si-1**	0.500						
**PU-1**	0.686						
**PU-2**	0.273						
**PU-2+CHSS**	0.273						
**PU-3+BZC-H**	0.043	0.068	0.08	0.043	0.080	x	0.043
**PU-4+NbMC**	0.225						

Platelets and coagulation

Remaining platelets

	Control loop	Si-1	PU-1	PU-2	PU-2+CHSS	PU-3+BZC-H	PU-4+NbMC
**Control loop**	x						
**Si-1**	0.005	x	0.047*	0.074	hemolysis	0.005	0.008
**PU-1**	0.005	higher	x	0.214	hemolysis	0.012*	0.260
**PU-2**	0.005	0.074	0.214	x	hemolysis	0.007*	0.028*
**PU-2+CHSS**	hemolysis						
**PU-3+BZC-H**	0.721						
**PU-4+NbMC**	0.066						

P-selectin MPs

	Control loop	Si-1	PU-1	PU-2	PU-2+CHSS	PU-3+BZC-H	PU-4+NbMC
**Control loop**	x						
**Si-1**	0.012	x	0.017*	0.161	0.208	0.012	0.012
**PU-1**	0.012	lower	x	0.208	lower	0.05*	1.00
**PU-2**	0.012	0.161	0.208	x		0.025*	0.327
**PU-2+CHSS**	0.012	0.208	0.036*	0.575	x	0.012	0.012
**PU-3+BZC-H**	0.208						
**PU-4+NbMC**	0.012	lower	1.00	0.327	lower	0.093	x

FXIIa activity

	No control loop	Si-1	PU-1	PU-2	PU-2+CHSS	PU-3+BZC-H	PU-4+NbMC
**Si-1**		x	0.386	0.005	0.005	0.005	0.005
**PU-1**		0.386	x	0.005	0.005	0.005	0.005
**PU-2**							
**PU-2+CHSS**							
**PU-3+BZC-H**							
**PU-4+NbMC**							

Prothrombin fragment F1+2

	Control loop	Si-1	PU-1	PU-2	PU-2+CHSS	PU-3+BZC-H	PU-4+NbMC
**Control loop**	x						
**Si-1**	0.028						
**PU-1**	0.63						
**PU-2**	0.028	No significant difference in generation of F1+2 prothrombin fragment F1+2 between the six materials (P = 0.071)					
**PU-2+CHSS**	0.075						
**PU-3+BZC-H**	0.075						
**PU-4+NbMC**	0.028						

TAT

	Control loop	Si-1	PU-1	PU-2	PU-2+CHSS	PU-3+BZC-H	PU-4+NbMC
**Control loop**	x						
**Si-1**	0.005	x	0.285	0.009*	0.508	0.028*	0.241
**PU-1**	0.005		x	0.005	0.005	0.005	0.005
**PU-2**	0.005	lower	lower	x	lower	lower	lower
**PU-2+CHSS**	0.005	0.508	lower	0.005	x	0.005	0.037*
**PU-3+BZC-H**	0.005	lower	lower	0.059	lower	x	0.386
**PU-4+NbMC**	0.005	0.241	lower	0.017*	lower	0.386	x

Results are shown as P values. Colors: light orange, significantly different from control loop; orange, significantly different from control loop and at least one other CVC material; red, significantly different from control loop and all other CVC materials. Because the dataset is small (n=10) a difference was only considered significant between materials if P < 0.05 and all ranks were pointing in same direction. * Ranks pointing in different directions.

Supplement 1b. Comparisons of the different catheter materials to the control loop and between the six different catheter materials.

The innate immune system

Complement activation by FXII or leukocytes

C3a anaphylatoxin

	Control loop	Si-1	PU-1	PU-2	PU-2+CHSS	PU-3+BZC-H	PU-4+NbMC
**Control loop**	x						
**Si-1**	0.047	x	0.203	0.203	0.139	0.508	0.241
**PU-1**	0.445						
**PU-2**	0.575			x			
**PU-2+CHSS**	0.007	0.139	0.005	0.007	x	0.005	0.005
**PU-3+BZC-H**	0.169						
**PU-4+NbMC**	0.114						

sC5b-9 marker

	Control loop	Si-1	PU-1	PU-2	PU-2+CHSS	PU-3+BZC-H	PU-4+NbMC
**Control loop**	x						
**Si-1**	0.005	x	0.285	0.169	0.114	0.169	0.114
**PU-1**	0.005	0.285	x	0.139	lower	0.799	0.878
**PU-2**	0.005	0.169	0.139	x	lower	0.241	0.445
**PU-2+CHSS**	0.005	0.114	0.005	0.005	x	0.005	0.005
**PU-3+BZC-H**	0.005	0.169	0.799	0.241		x	0.721
**PU-4+NbMC**	0.005	0.114	0.878	0.445		0.721	x

Acute immune reaction: cytokine and growth factors released from leukocytes/platelets

Interleukin 8

	Control loop	Si-1	PU-1	PU-2	PU-2+CHSS	PU-3+BZC-H	PU-4+NbMC
**Control loop**	x						
**Si-1**	0.037	x	0.203	0.508	0.114	0.005**	0.575
**PU-1**	0.074		x				
**PU-2**	0.047	0.508	0.333	x	lower	0.005**	0.575
**PU-2+CHSS**	0.005	0.114	0.005**	0.009*	x	0.005**	0.005
**PU-3+BZC-H**	0.575						
**PU-4+NbMC**	0.005	0.575	0.169	0.575	lower	0.007*	x

TNF-α

	Control loop	Si-1	PU-1	PU-2	PU-2+CHSS	PU-3+BZC-H	PU-4+NbMC
**Control loop**	x				0.005		
**Si-1**	0.646						
**PU-1**	0.241						
**PU-2**	0.646						
**PU-2+CHSS**	0.005	0.005	0.005	0.005	x	0.007	0.005
**PU-3+BZC-H**	0.575						
**PU-4+NbMC**	0.508						

VEGF

	Control loop	Si-1	PU-1	PU-2	PU-2+CHSS	PU-3+BZC-H	PU-4+NbMC
**Control loop**	x						
**Si-1**	0.018	x	0.012*	0.018	0.401	0.012**	0.012**
**PU-1**	0.176		x	0.866		0.93	0.575
**PU-2**	0.018	lower	0.866	x	0.091	0.018**	0.237
**PU-2+CHSS**	0.043	0.401	0.036*	0.091	x	0.025*	0.025*
**PU-3+BZC-H**	0.866					x	0.093
**PU-4+NbMC**	0.091						x

Results are shown as P values. Colors: light orange, significantly different from control loop; orange, significantly different from control loop and at least one other CVC material; red, significantly different from control loop and all other CVC materials. Because the dataset is small (n=10) a difference was only considered significant between materials if P < 0.05 and all ranks were pointing in same direction. * Ranks pointing in different directions. **only significant with material not different from the control loop.
